# Abdominal Sarcoidosis: A Mystery Revisited

**DOI:** 10.1002/ccr3.71557

**Published:** 2025-12-09

**Authors:** Lovenish Bains, Nidhi Paswan, Soukat Ali Khan, Shramana Mandal

**Affiliations:** ^1^ Department of General Surgery Maulana Azad Medical College New Delhi India; ^2^ Department of Pathology Maulana Azad Medical College New Delhi India

**Keywords:** abdominal sarcoidosis, granulomatosis, hepatic, laparoscopy, peritoneal, splenic

## Abstract

Unexplained lymphadenopathy is an often‐encountered symptom or abnormal finding during physical examinations in practice, and it can stem from a wide range of causes. These require multiple investigations to reach a diagnosis. One of the rare causes is Sarcoidosis, which is a multi‐system disease characterized by the infiltration of various organs by non‐necrotizing granulomas with intrathoracic involvement being the most common presentation. It has a global prevalence of approximately 5–64 cases per 100,000 people, depending on the region. Abdominal sarcoidosis accounts for approximately 30% of extrapulmonary manifestations. Its diagnosis presents significant challenges. We describe a case of abdominal sarcoidosis and the role of laparoscopy in diagnosis. A 44‐year‐old woman presented with 6 months of easy fatigability and dull, aching abdominal pain. She also experienced occasional fever but had no history of tuberculosis exposure or associated symptoms. The general examination was unremarkable. Abdominal examination revealed splenomegaly. Ultrasound and subsequent computed tomography showed multiple enlarged lymph nodes (LNs) in the periportal, peripancreatic, mesenteric, and retroperitoneal regions, along with splenomegaly. Chest imaging revealed no significant findings. Differential diagnosis, including tuberculosis and lymphoma, was initially considered. An EUS‐guided biopsy of the abdominal LNs showed features of granulomatous lymphadenitis. However, the diagnosis remained unclear, prompting an FDG‐PET scan, which revealed findings consistent with those seen on previous imaging. Diagnostic laparoscopy was performed with periportal and peripancreatic LN sampling, along with an edge biopsy of the liver. Intraoperatively, multiple nodular hepatic and splenic lesions were identified, along with small, waxy nodules on the parietal peritoneum and enlarged periportal and peripancreatic LNs. Histopathological examination report revealed large non‐caseating granulomatous lesions containing epithelioid cells, suggesting sarcoidosis. Intra‐abdominal sarcoidosis, though rare, often presents asymptomatically, making it difficult to diagnose. Its clinical and radiological features are nonspecific and can mimic neoplastic or infectious diseases. In endemic regions of tuberculosis, its presentation can pose significant diagnostic challenges. Diagnosis relies on clinicopathologic findings and the exclusion of other granulomatous and autoimmune diseases. Diagnostic laparoscopy is required to reveal the involvement of granulomatous disease in the visceral and parietal peritoneum and reach a histopathological diagnosis. Especially in cases of isolated abdominal sarcoidosis, diagnostic laparoscopy played an important role in forming a definitive diagnosis.

## Key Clinical Message

Abdominal sarcoidosis is rare and poses significant diagnostic challenges due to its nonspecific symptoms and radiologic resemblance to malignancy or infections. It mimics diseases like tuberculosis or carcinoma. Laparoscopy plays a crucial role in obtaining a definitive histopathological diagnosis, minimizing morbidity, and enabling individualized patient management.

**Abbreviations**
ACE: angiotensin converting enzyme
ALP: alkaline phosphatase
ALT: alanine aminotransferase
AST: aspartate aminotransferase
CECT W/A: contrast‐enhanced computed tomography—whole abdomen
CT: computed tomography
EUS: endoscopic ultrasonography
FDG: fluorodeoxyglucose
HPE: histopathology
LN: lymph node
PET: positron emission tomography
PTH: parathyroid hormone
USG: ultrasonography
VIT D: vitamin D

## 1 Introduction

Extrapulmonary involvement in sarcoidosis occurs in about 25%–50% of patients [1], and the abdomen is the most common extrapulmonary site. Abdominal LN involvement is observed in approximately 30% of sarcoidosis patients, while extensive LN enlargement (node size > 2 cm and involvement of four or more sites) in the abdomen is observed in roughly 10% of patients [2]. Abdominal lymphadenopathy is a frequent clinical finding and can result from various causes, such as tuberculosis, malignancy, carcinoma, or fungal infections. Sarcoidosis is a rare but important cause, with intrathoracic disease being the most common manifestation [3]. Sarcoidosis is a disease that can affect multiple organ systems and is characterized by the presence of non‐necrotizing granulomas in affected tissues. Although its exact cause remains unknown, the condition was first reported by Besnier in 1889. Sarcoidosis can develop in individuals of any age or ethnicity, but it is most commonly diagnosed in people between 25 and 40 years old, accounting for approximately 70% of cases at the time of presentation [4]. According to recent Swedish national registry data, the annual incidence of sarcoidosis is estimated at 11.5 per 100,000 individuals, with a prevalence rate of 0.16% [5]. The disease appears to be less common among Hispanics and Asians, with incidence rates ranging from 3 to 4 per 100,000 and a prevalence as low as 0.02% [6]. In India, study at Vallabhbhai Patel Chest Institute (VPCI), Delhi, estimated the prevalence of sarcoidosis at 61.2 per 100,000 new cases annually [7]. Because abdominal sarcoidosis is uncommon and its symptoms are often nonspecific, diagnosis is frequently delayed or missed. Patients may present with vague abdominal discomfort, weight loss, or hepatosplenomegaly. Diagnosing abdominal sarcoidosis is challenging and often requires excluding other potential causes. If left unrecognized, this condition can lead to significant health complications and even increased mortality. We describe a case of abdominal sarcoidosis that was challenging to diagnose because of the wide range of differential diagnosis and the role of Diagnostic Laparoscopy in the workup of abdominal sarcoidosis.

## 2 Case Summary

A 44‐year‐old lady presented with generalized, dull aching pain in the abdomen for 6 months. Pain was insidious in onset, dull aching in nature, non‐progressive, non‐radiating, and had no aggravating or relieving factors. There was no significant history of fever, weight loss, decreased appetite, vomiting, fatigability, or any skin changes. No history of tuberculosis or any contact with a person having tuberculosis. On clinical examination, the patient had cervical lymphadenopathy measuring ~1 × 1 cm over the right posterior triangle of the neck (level 5). Abdominal examination revealed splenomegaly (5 cm below the left costal cartilage).

## 3 Investigations

Laboratory investigations revealed mild anemia (Hb 9.5 g/dL, normal: 12–15 g/dL), elevated liver enzymes (AST 32 U/L [normal: 5–40 U/L]; ALT 46 U/L [normal: 5–40 U/L]; ALP 156 U/L [normal: 44–147 U/L]), and hypercalcemia (11.2 mg/dL, normal: 8.5–10.5 mg/dL). Vitamin D was 28.4 IU (normal: 30–100 IU), parathyroid hormone was 8.27 pg/mL (normal: 13.6–85.8 pg/mL). Complete blood count showed: Total leukocyte count 5600/μL (normal: 4000–11,000/μL), differential leukocyte count showed neutrophils 58% (normal: 50%–70%), lymphocytes 31% (normal: 20%–40%), monocytes 9% (normal: 2%–8%), eosinophils 2% (normal: 1%–4%), and platelet count (198 × 10^9^/L). No thrombocytopenia was observed.

Tuberculosis workup came negative (sputum for acid‐fast bacilli/cartridge‐based nucleic acid amplification test) was negative, Tuberculin test/Purified Protein Derivative test showed 5 mm induration. Chest X‐Ray was normal and showed no evidence of tuberculosis or any hilar lymphadenopathy.

## 4 Differential Diagnosis

**FIGURE 1:**
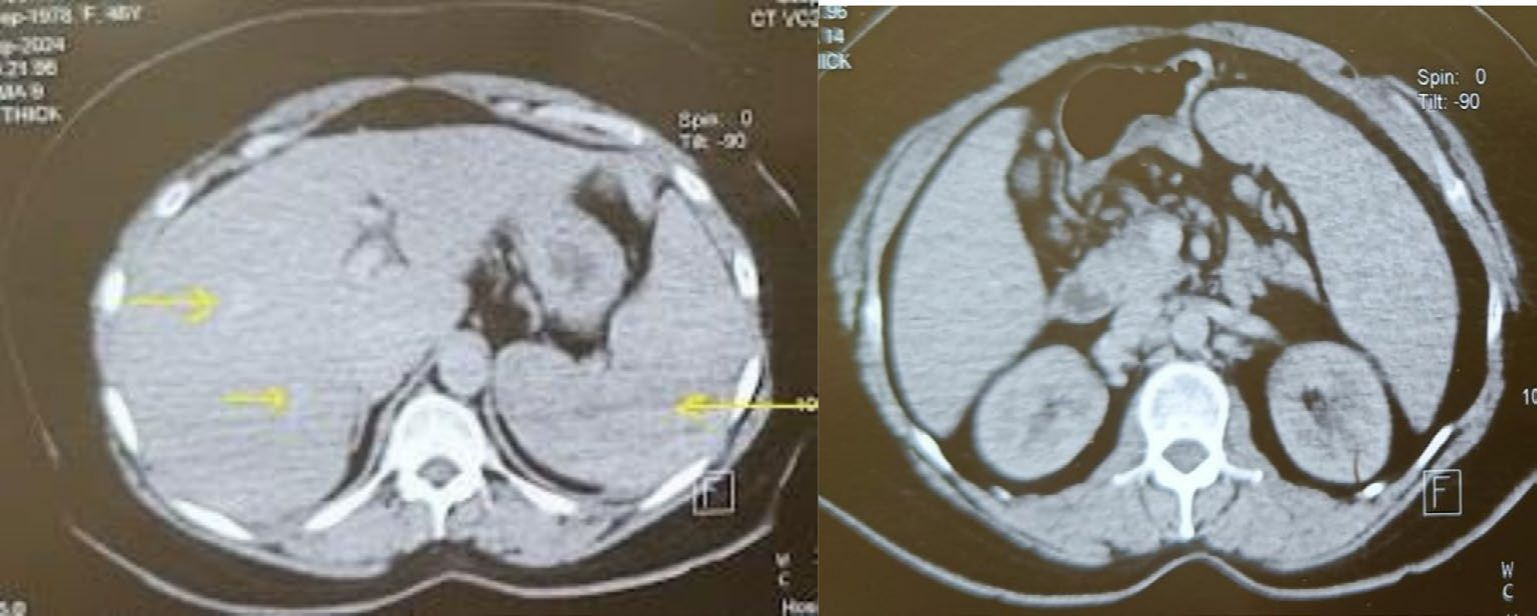
Transverse section on CT showing nodular macro deposits over liver and spleen with abdominal lymphadenopathy (yellow arrows—nodules better appreciated on PET‐CT).

**FIGURE 2:**
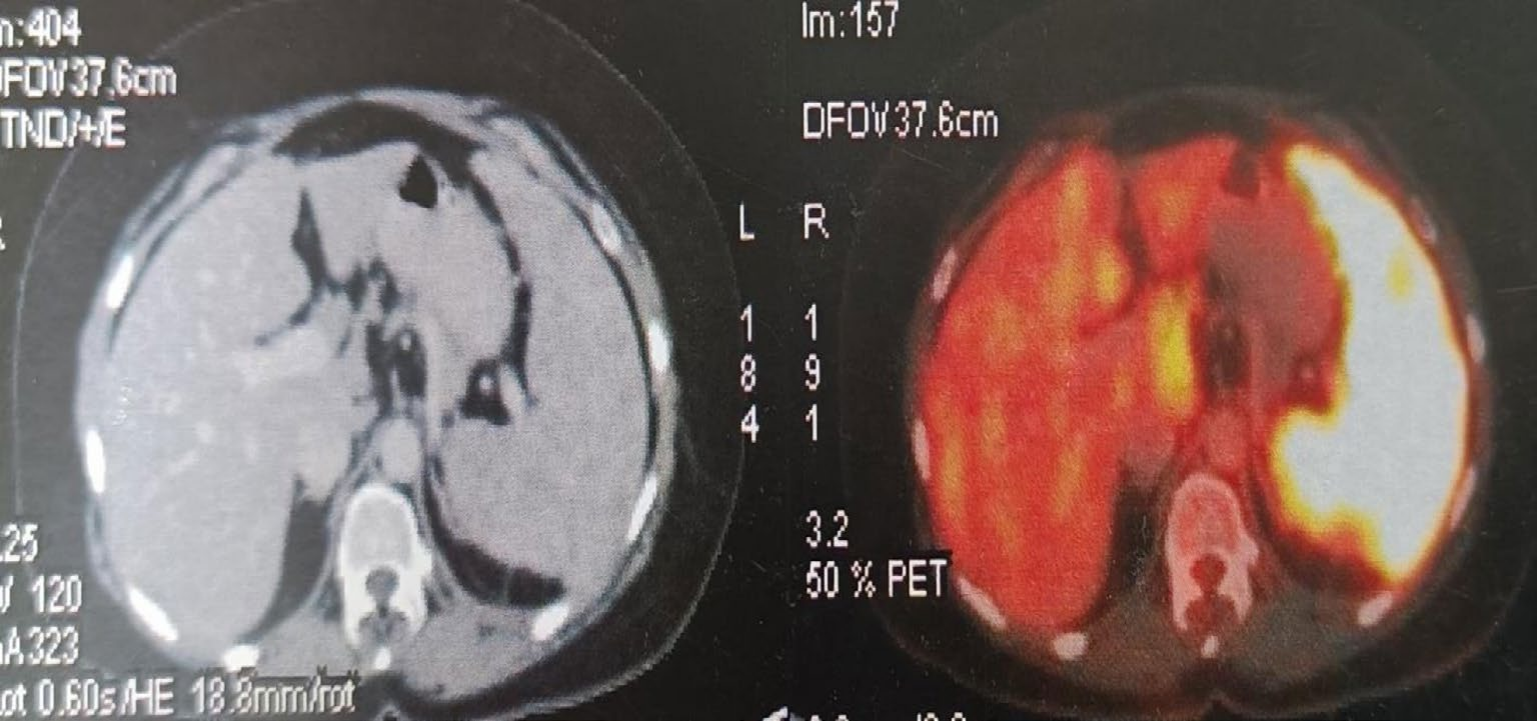
Transverse section on PET‐CT showing increased FDG uptake in liver, spleen, and multiple abdominopelvic lymph nodes.

**FIGURE 3:**
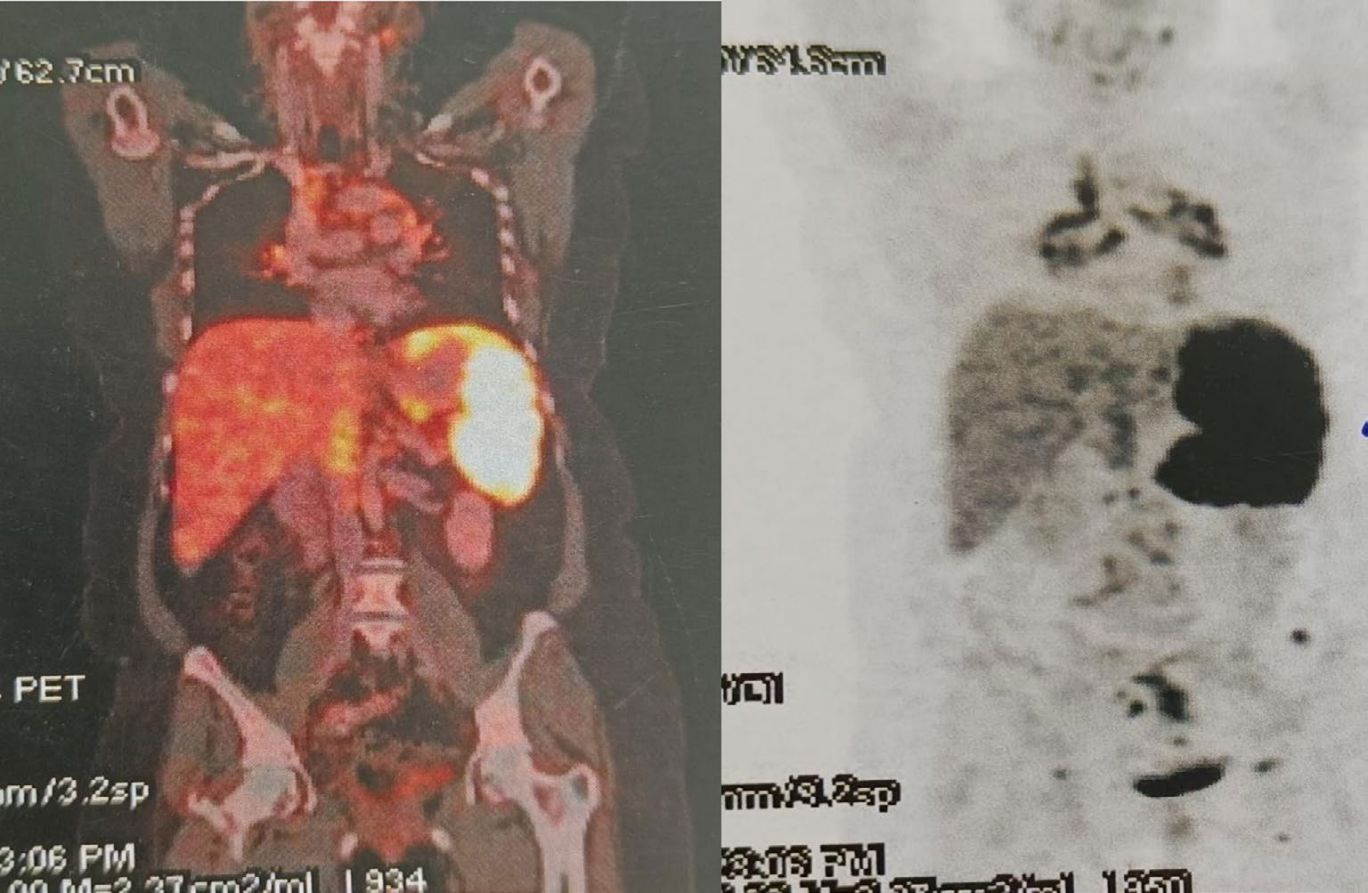
Coronal section on PET‐CT showing increased FDG uptake in liver; spleen; and periportal, perihepatic, para‐aortic lymph nodes.

Key differentials kept in mind were tuberculosis, fungal infections, lymphoma, autoimmune disorders. A comprehensive workup combining imaging, histopathology, and clinical correlation is critical. The patient presented to us with all the investigations and was unable to reach a definitive diagnosis. Abdominal ultrasonography revealed a mildly enlarged liver with normal echotexture. Multiple hypoechoic, well‐defined nodules were seen throughout the hepatic parenchyma, the largest measuring 1–3 cm. No intrahepatic biliary dilatation was noted. The spleen was enlarged (measuring 14 cm, normal: < 12 cm). Multiple small, hypoechoic nodules were scattered throughout the splenic parenchyma. Contrast‐enhanced computed tomography (Figure 1) and positron emission tomography‐computed tomography (PET‐CT) (Figure 2) showed hepatosplenomegaly with multiple hypodense nodules in the liver and spleen. Extensive retroperitoneal and mesenteric lymphadenopathy was present. Fluorodeoxyglucose‐avid uptake was seen in discrete and conglomerated multiple cervical, mediastinal, periportal, portacaval, gastrohepatic, aortocaval, peripancreatic, retrocaval, paraaortic, and mesenteric LNs, the largest ~3.7 × 1.9 cm. Diffusely fluorodeoxyglucose uptake was also seen in mild to moderate splenomegaly likely to represent extra lymphomatous distant lesions, SUV max 8.0 (maximum standardized uptake value) (Figure 3). CT and PET‐CT were likely suggestive of metabolically active lymphoma. Endoscopic ultrasonography‐guided abdominal LN biopsy was performed which was suggestive of granulomatous lymphadenitis.

**FIGURE 4:**
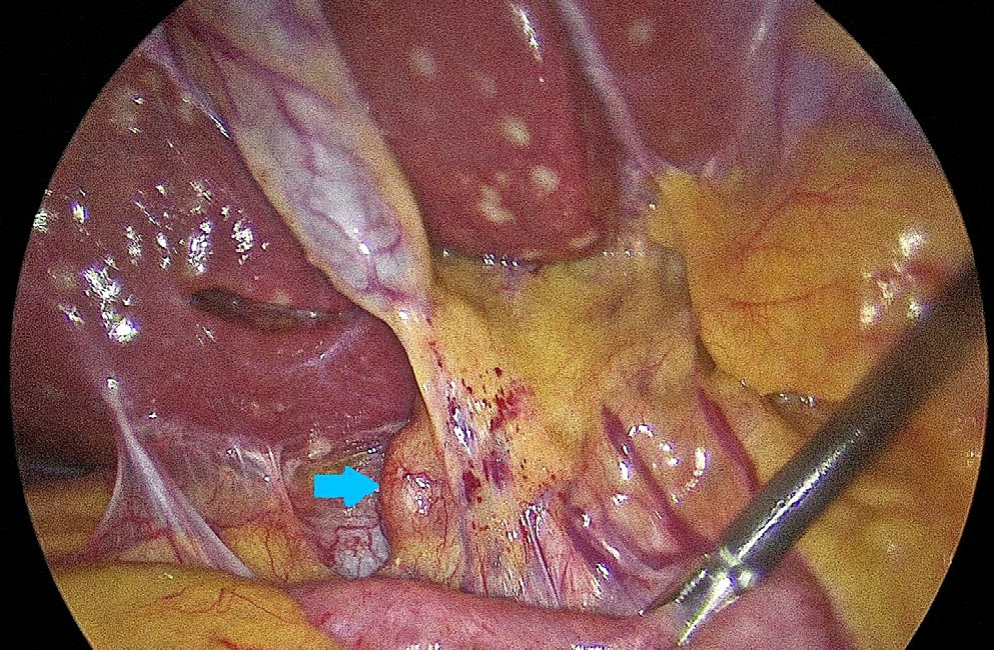
Multiple nodules over liver and parietal peritoneum with enlarged periportal lymph nodes (blue arrow).

**FIGURE 5:**
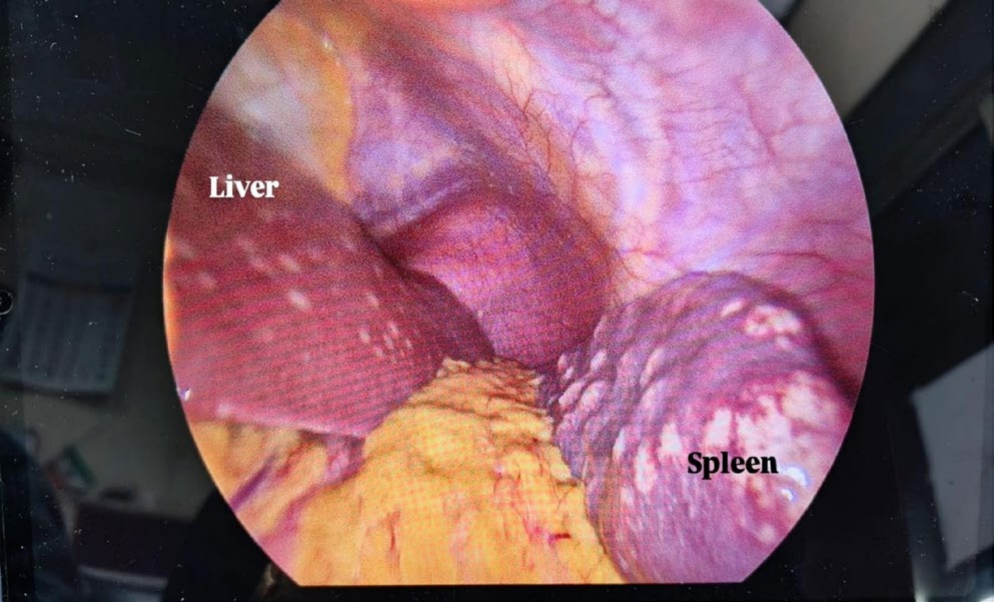
Multiple macronodules seen over liver and spleen.

**FIGURE 6:**
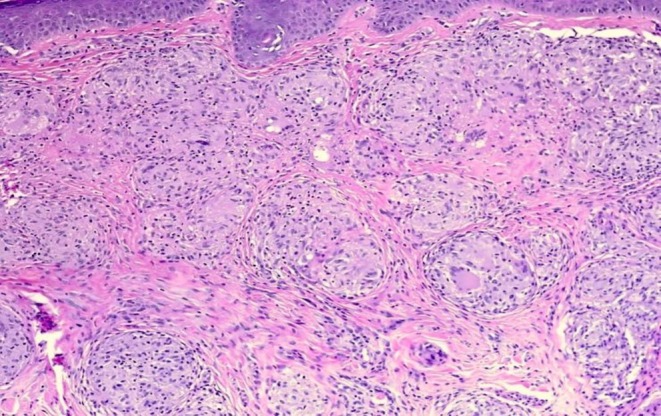
HPE showing granuloma with epithelioid cells with surrounding lymphocytes (H&E ×100).

Due to the inconclusiveness of the above‐mentioned findings, malignancy could not be ruled out. Therefore, the patient underwent diagnostic laparoscopy, where periportal and peripancreatic LN sampling with biopsy of the liver and sampling of peritoneal nodules was done. Intraoperatively, multiple flat yellow‐colored nodular hepatic and splenic lesions were identified measuring from 1 cm to the largest being 4 cm with small pinpoint waxy nodules over the parietal peritoneum and multiple enlarged periportal and peripancreatic LNs (Figures 4 and 5). The histopathology report showed granulomatous lesions with multiple epithelioid cells and cuffing of lymphocytes, with occasional asteroid bodies noted. Non‐caseating granulomas were present and negative for acid‐fast bacilli. Features were suggestive of abdominal sarcoidosis (Figure 6).

## 5 Result/Treatment

Due to the possibility of granulomatous disease and excluding Tuberculosis, serum angiotensis converting enzyme levels were done which came out to be elevated (> 140 mmol/L), adding to the suspicion for Sarcoidosis. The anemia observed in this patient was likely secondary to the chronic inflammatory process associated with sarcoidosis rather than bone marrow involvement, as evidenced by the absence of other cytopenias and the normocytic, normochromic nature of the anemia. The post‐operative period was uneventful. The patient was initiated on corticosteroid therapy 70 mg/day for 8 weeks, which was subsequently tapered to 30 mg/day over a period of 4 months (10 mg/day reduction every month). The patient was discharged and was kept on close follow‐up. The patient is symptomatically better and doing well. Serum angiotensin converting enzyme levels were done which decreased from > 140 to 34 mmol/L over a period of 3 months.

## 6 Discussion

Sarcoidosis is a chronic inflammatory condition that can affect multiple organ systems, with its cause still not fully understood. It is defined by the formation of noncaseating granulomas clusters of immune cells, in various tissues and is diagnosed after excluding other granulomatous diseases such as tuberculosis, fungal infections, and autoimmune disorders [8]. The prevalence of sarcoidosis varies widely across populations, with global rates estimated between 2 and 60 per 100,000 individuals [9]. The disease is most commonly identified in adults aged 20–40 years. Regarding gender distribution, studies have shown mixed results, with some reporting slight female predominance while others show no significant gender difference or even slight male predominance, depending on the population studied [5].

Extrapulmonary involvement is reported in 25%–50% of patients [10], and the abdomen is one of the most common extrapulmonary sites. Among patients with extrapulmonary involvement, abdominal manifestations occur in a significant proportion of cases. The liver and spleen are the most common abdominal sites of involvement, with hepatic involvement occurring in 50%–80% of cases and splenic involvement in 40%–80% of cases. Lymph node involvement occurs in approximately 30% of patients, and kidney involvement is less frequent. These percentages vary significantly between autopsy findings and clinical manifestations, with autopsy studies typically showing higher rates of involvement than clinically apparent disease [11].

Sarcoidosis most frequently affects the lungs, with about 90% of patients showing pulmonary involvement, and the lymphatic system, which is affected in around 30% of cases. Cardiopulmonary involvement is the main cause of death [11]. Abdominal sarcoidosis is relatively rare and typically does not produce symptoms, making it difficult to identify. Its clinical presentation and imaging findings are often non‐specific and can resemble those seen in cancer or infectious diseases. This diagnostic challenge is heightened in regions where tuberculosis is prevalent, due to overlapping features. The liver and spleen are the abdominal organs most often affected, and they may develop nodular lesions. When comparing LN sizes, those found in sarcoidosis are generally smaller than those seen in lymphoma (averaging 2.6 ± 1.7 cm in sarcoidosis versus 8.0 ± 5.5 cm in lymphoma), though in some cases, sarcoidosis‐related nodes can be as large as 7.5 cm [2].

Clinical signs of abdominal sarcoidosis may include liver enlargement, persistent fatigue, discomfort or pain in the right upper abdomen sometimes accompanied by itching (seen in about 5%–15% of cases), as well as fever, jaundice, and, less commonly, weight loss (reported in fewer than 5% of patients) [12]. Splenic involvement is typically identified through imaging studies rather than through symptoms or laboratory findings, and it occurs at a rate similar to that of liver involvement. Some patients may present with general symptoms and significant splenic enlargement, which is observed in up to 6% of cases [13]. Enlargement of abdominal LNs is found in approximately 30% of individuals with sarcoidosis, most often in areas such as the hepatic hilum, para‐aortic and celiac regions, around the iliac vessels, or within the mesentery. These LNs generally appear as hypodense lesions on imaging, usually measuring between 1 and 2 cm in diameter. In up to 10% of cases, LNs may exceed 2 cm, which can make it challenging to distinguish sarcoidosis from malignant conditions like lymphoma [2].

Suspicion of liver involvement in sarcoidosis may arise when laboratory tests reveal abnormal results. Notably, increased levels of alkaline phosphatase and gamma‐glutamyl transferase are often associated with cholestasis and hepatic sarcoidosis, and these abnormalities can be significant indicators of liver involvement. Mild elevations in alanine aminotransferase (ALT) and aspartate aminotransferase (AST) are also observed, occurring in about half of asymptomatic individuals with hepatic sarcoidosis. Additionally, hyperglobulinemia is a frequent finding in these patients. Serum angiotensin‐converting enzyme (ACE) concentrations are elevated in a substantial proportion of those with active sarcoidosis‐reported in up to 60%–80%‐but are less commonly raised in chronic cases or in individuals receiving corticosteroid therapy. While a normal ACE level does not exclude the diagnosis, an elevated ACE can help differentiate sarcoidosis from other granulomatous diseases when considered alongside clinical and laboratory findings [14, 15]. Liver abnormalities are detected by CT or ultrasound in approximately 50% of patients, with hypoattenuating nodules (5%–35%) and hepatomegaly (8%–18%) being the most common findings [15, 16]. Splenic involvement may manifest as enlargement or nodules (> 1 cm) in up to 15% of abdominal CT scans, often appearing as irregular, hypodense lesions with a tendency to coalesce. Calcifications (punctate or well‐defined) are observed in 16% of cases. MRI and 18F‐FDG PET/CT offer superior resolution for identifying hepatic and splenic nodules, which typically appear hypointense on T2‐weighted MRI and show variable FDG avidity. FDG uptake in sarcoidosis is nonspecific in both intensity and pattern and marked FDG uptake in LNs and parenchymal organs can mimic malignancy, specifically lymphoma and diffuse metastatic disease. Despite this nonspecificity, FDG uptake can decrease when sarcoidosis is treated, and PET can be useful in monitoring the effectiveness of therapy [17].

Previously, obtaining a tissue diagnosis in patients with mesenteric or retroperitoneal lymphadenopathy required an open surgical procedure (laparotomy). Today, laparoscopy has become a valuable alternative, providing a less invasive approach that reduces the risks and recovery time associated with open surgery. Laparoscopy allows for direct visualization and sampling of abdominal LNs and organs, making it especially useful when needle biopsies are inconclusive or not feasible. Case reports have demonstrated the utility of laparoscopy in differentiating sarcoidosis from other serious conditions. For example, one patient with peritoneal nodules initially thought to be metastatic cancer was ultimately diagnosed with sarcoidosis through laparoscopic biopsy [18]. Similarly, in cases of isolated splenic sarcoidosis where imaging could not distinguish between sarcoidosis and other diseases, laparoscopic splenectomy provided both diagnostic clarity and a minimally invasive treatment option [19].

The current case highlights the importance of diagnostic laparoscopy in evaluating intra‐abdominal lesions. This procedure is both effective and safe for obtaining tissue samples when non‐invasive methods do not yield a diagnosis. In summary, laparoscopy offers a minimally invasive, reliable method for diagnosing and managing abdominal sarcoidosis and is particularly valuable when less invasive tests are inconclusive or when therapeutic intervention is required.

## 7 Conclusion

Isolated abdominal sarcoidosis often presents with vague symptoms making it difficult to diagnose. Its clinical and radiological features are nonspecific and can mimic neoplastic or infectious diseases. Abdominal sarcoidosis requires a multidisciplinary approach to overcome diagnostic and management challenges. Laparoscopy has evolved as a minimally invasive procedure to obtain a definitive histopathological diagnosis, further reducing the morbidity of open surgery. It has also helped to access areas that were not possible to reach with needle biopsy. This case report states the pivotal role of laparoscopic surgery in diagnosing abdominal sarcoidosis and highlights the importance of individualized patient care.

## Author Contributions

**Lovenish Bains:** conceptualization, formal analysis, methodology, project administration, resources, supervision, validation, visualization, writing – review and editing. **Nidhi Paswan:** conceptualization, data curation, formal analysis, investigation, methodology, writing – original draft, writing – review and editing. **Soukat Ali Khan:** data curation, investigation, methodology, resources, validation, writing – review and editing. **Shramana Mandal:** investigation, resources.

## Funding

The authors have nothing to report.

## Ethics Statement

The authors have nothing to report.

## Consent

Written consent for the publication of this case report was obtained from the patient. Written informed consent for the publication of this case report and for the accompanying images was obtained from the patient. A copy of the written consent is available for review by the Editor‐in‐Chief of this journal.

## Conflicts of Interest

The authors declare no conflicts of interest.

## Data Availability

All data generated or analyzed during this study are included in this published article.
